# Notes and Comments

**Published:** 1924-07

**Authors:** 


### NOTES AND COMMENTS.

### MR. WHEATLEY'S HOUSING BILL.

THE remarkable Housing Bill produced by the Minister of Health makes exceedingly slow progress, and it will be as much as the Government can do to get it through Parliament before the Autumn Session. The delay is cause for gratification rather than regret, and we hope that the powerful critical batteries that are being turned upon the Bill will produce substantial modifications. The only point of view from which the measure concerns us in this place is that of the good housing of the people and the influence of that housing upon the national health. From this standpoint it is entirely unnecessary. There is not the smallest reason to suppose that it will increase the number of houses already being produced by Mr. Neville Chamberlain's Act, and Mr. Wheatley has been wise and wary enough to let that Act run its all too short course, side by side with his own legislation. The houses that it does yield will be infinitely more costly in the end than those that are at present being built. Under the 1923 Act 1,000 houses can be subsidised for £100,000 at a charge of a half-penny in the pound on the rates for twenty years. Under Mr. Wheatley's scheme the subsidies would amount to nearly £500,000, with a charge of three half-pence on the rates for forty years certain, while beyond that period it would be difficult to obtain the full rents, to say nothing of the certainty of the allowance for repairs being insufficient. This grandiose plan for building two and a-half million houses in fifteen years—a far larger number than we need—has now been modified in the sense that it is to be subject to review at the end of every three years. This very necessary precaution may not improbably involve its painless extinction at no very distant date.

### THE MENTALLY DEFECTIVE.

HOW many of us realise that ½ per cent. of the population of these islands is mentally defective? One abnormal person in two hundred may not seem very much, yet the total works out at 150,000, many of whom marry and produce another generation perhaps even more defective, while all of them are a burden to themselves and a drag upon the community. These serious facts provided abundant material for discussion at the recent Conference organised by the Association for Mental Welfare. The Minister of Health, who made an interesting speech, expressed his readiness to give substantial help to the efforts that are being made for the improvement of a lamentable situation. It is a situation that requires much more careful consideration than it has yet received, and one of the first needs is to get the country to understand the extent of the trouble and what is being, and ought yet to be, done to alleviate it. Mental defect readily degenerates into actual insanity, which we are bound to do all that is possible to prevent. No small proportion of the crime of the country is the result of wits so defective that they cannot effectually discriminate between right and wrong. Vigilance may save the worst results, but we must begin at the beginning if we are to achieve real success. The mentally unfit must be prevented from reproducing their kind and so compelling society to go round a vicious circle. But let us not forget that the squalid conditions of slum life have unquestionably a tendency to produce that weakness of mind which accounts for so much of the crime committed by the ill-educated and ill-housed. To reduce slums means to reduce crime by improving health, strengthening character, increasing self-respect and lessening the volume of feeble-mindedness.

### THE CINEMA IN EDUCATION.

FOR the last three years an amateur Cinema Commission has been inquiring into the influence of the cinema upon young people. It has already reported upon its physical and moral influences and is now preparing a report on its educational possibilities. We gather from a letter in the newspapers from the Rev. James Marchant, the secretary, that the Commission finds that those possibilities are very great, especially in the direction of what we may call Imperial teaching. The film in the school may obviously be of very great value. It can teach the simple rules of health and can at least attempt to interest school children in really useful knowledge. There is nothing so romantic as facts properly handled, and the British Empire is a much more romantic affair than the sickly and disjointed dramas and "knockabout" farces which form the staple of the cinema. The conquest and gradual building up of the Empire makes an enthralling story; so does the Empire's war upon disease for the benefit of the peoples who have come beneath its sway. We know to-day that mental and physical health are more closely allied than was formerly supposed, and neither of them is improved by constant looking at vacuous spectacles. In the interests of both, therefore, we hope that when the new report of the Cinema Commission is issued, it will stimulate the Educational Authorities, central and local, to provide opportunities for one of the most valuable and fruitful uses of the cinema.

### RAILWAY STRIKES AND HEALTH.

AMID all the talk about the wanton selfishness of the recent strike of certain of the electrical workers on the London tube railways, and of the injury they have caused to the trade union system, we have been in danger of losing sight of a peril which must have been painfully obvious to many of those who suffered daily inconvenience. The effect upon elderly people and women of standing for half-an-hour or more twice a day in a railway carriage so tightly packed that even strap-hanging is impossible, breathing hot and vitiated air, and being squeezed a little more tightly at every stopping-place is, at the best, unhappy. At the worst it may be quite serious. People with weak hearts or in poor health, who were fatigued at the start from a day's work, have been known to reach home in a condition of serious distress and suffering from a degree of exhaustion from which they have hardly recovered by the time the next day's squeeze arrives. People, young or old, who work in great towns have become inured to strap-hanging and similar inconveniences, but they may be excused if they feel bitterly resentful towards men who, from selfishness in the crudest form, subject them to acute discomfort daily for a week or two at a time.

### AN ANNUAL OVERHAUL.

THE annual medical overhaul which was, we believe, initiated by a great American Assurance Company, is making definite, albeit slow, progress in England. A year ago the Wesleyan and General Assurance Society began to examine such of its larger policy-holders as were willing to be overhauled, and it has now extended the plan to the smaller ones. The results of the examinations, which are made by the Company's medical officer, are divulged only to the policy-holder, who, it would appear, is, generally speaking, grateful. The advantage of these examinations is that they afford opportunities for detecting incipient disease and enable ameliorative steps to be taken while yet there is time. It is obviously to the advantage of the Assurance Companies to keep their policy-holders alive as long as possible, and, in so doing, they are helping in the great work of preventing disease. It is even more obviously important to the individual who believes himself to be sound and fit, either to have his belief verified or to be warned that care in this or that direction is desirable, or, it may be, imperatively necessary.

### CARE OF THE CONSUMPTIVE.

THE National Association for the Prevention of Tuberculosis are holding their tenth annual conference on July 3 and 4. Their programme includes a visit to the Burrow Hill training colony at Frimley. This will form an interesting prelude to the discussion which is to take place on the part played by training colonies in the treatment of tuberculosis. When the members of the conference turn from this subject to that of the organisation of Care Committees, they will be concerning themselves with a matter less picturesque, but of greater scope and importance. If they succeed in the light of their experience in hammering out some uniform lines of procedure and in defining their sphere of activity, much will have been accomplished. The successful working of these Committees will remain dependent, as heretofore, on the sincerity and tact of the individual workers. They must be well informed on the subjects which they carry into the homes of the patients, and they must avoid fussiness and inquisitorial methods. They have on the one hand to carry persuasion to the patient and his family, and on the other to avoid interference with the family doctor and the Medical Officer. They have indeed a difficult and, at the same time, a most important task, for the undertaking of which they deserve the thanks of the community.

### HOSPITALS AND HISTORY.

TO take parties of visitors, *moyennant finance*, to places of interest in London for the benefit of King Edward's Hospital Fund was a brilliant idea. It has been carried out successfully during the last few weeks, and the personally-conducted parties have usually been fairly numerous. There are obvious limits to their size, since they would lose their attraction, as well as their convenience, if the visitors experienced difficulty in following the historical and descriptive particulars given by their guides. There is no more admirable way of teaching history than telling the story of a building or a site on the spot, and the many Britons from overseas, to say nothing of Americans, who have been imbibing English history in this agreeable way would, no doubt, be glad of further opportunities of the kind. The idea might, perhaps, be extended for the benefit of hospitals in other parts of the country. Every county contains buildings that are much visited for the sake of their beauty and their interest, human or historic, and the Voluntary Hospital Committees might sometimes turn an honest penny in this way. The mere fact that the visitors' fees were to help hospital funds would be an inducement to many people to join circles of this kind. The average Briton knows so little of the history of his own country that it would almost be worth while to pay him to learn it, but if we can induce him to pay for the acquisition of this desirable class of knowledge, we are keeping more clearly within the bounds of the fitness of things.

### THE SEASONS AND SUICIDE.

SOME interesting statistical researches have recently been carried out in Finland by Dr. R. Ehrström, who started with the hypothesis that spring lassitude is a neurosis—a psychopathic trait indicating lack of nervous stability and tone in its possessor. If this hypothesis be correct, one would expect to find other manifestations of neuroses to be more frequent in the spring than at any other season in the year. And this is what Dr. Ehrström has succeeded in demonstrating. During 1921 and 1922 there were 3,039 persons attending his hospital for symptoms which were wholly, or mostly, indicative of a neurosis. The first attendances of these persons were classified according to the months in which they took place, and it was found that these first attendances were most frequent in the spring and early summer. After June they began to decline, and this decline was maintained more or less steadily for the rest of the year, their number being smallest in December. Dr. Ehrström's suicide curve was obtained by classifying, according to the months in which they occurred, all the 5,471 suicides committed in Finland in the period 1851–1907. This curve was remarkably like the neurosis curve ; there was a rise during the spring and early summer, and a fall from June to December. The explanation of these curves is, in Dr. Ehrström's opinion, to be sought in the one word Vitamins. The supply of these accessory food substances is greatest in the summer, which in the Northern European countries is very slow in coming. From midsummer to autumn the body accumulates a store of vitamins, and it enjoys the benefits of this store throughout the first half of the winter. Not until the spring does the exhaustion of the accumulation make itself felt, and during the first months of the summer the human body is still suffering from the inadequacy of its vitamin supply in the earlier months of the year. So now we know why we are tired in the spring, and why the autumn finds us cutting the capers which we associate with the "feeling" supposed to be generated by certain well-advertised salts.

### THE GLASGOW CRIME.

THE famous Lister Ward of the Glasgow Infirmary has been well and truly murdered "according to plan." No more wanton deed of destruction in time of peace occurs to the memory, and future historians of Glasgow will for ever lament the blindness and obstinacy of a small number of its irresponsible citizens in the twentieth century. But this is no mere question of Glasgow history. The preservation of the room in which Lord Lister did such magnificent work for humanity was a matter for the whole world, and the whole world joined in imploring those with whom its fate rested to preserve so precious a relic. Mr. James Morris, who strove so valiantly to save the ward, has described it most truly as "a shrine of inestimable spiritual value." It was because it was such a shrine that America offered to find the money that would have kept it in being; but, indeed, the money could have been found in Glasgow itself, whose own principal newspaper declared that the city's honour was at stake. But the managers were deaf as Ailsa Craig. Every reason they gave for destruction was controverted, and even belied by their own action; yet they persisted with obstinate stubbornness to the bitter end. It is long since the first censure was uttered upon the remover of landmarks, and it will be almost as long before the last is uttered upon the stiff-necked generation which has razed the Lister Ward from the face of the earth.

### FALLING DEATH RATE FROM DIABETES.

ONE of the few benefits conferred by the Great War was an appreciable decline in the incidence of diabetes in those countries in which dietetic restrictions were imposed wholesale. Soon afterwards, the return to a more generous dietary was accompanied by a rise in the frequency of diabetes, and it is probable that many of these after-war cases would have developed before the determination of hostilities but for the lean years of the Great War. Since then insulin has been discovered, and it is interesting to note its effect on the death-rate from diabetes. That enterprising and scientifically minded American institution, the Metropolitan Life Insurance Company, has recently issued a report according to which there is a small, but probably significant, decline of 6.4 per cent. in the death-rate from diabetes among its industrial policy-holders. The decline is the more significant since in the preceding three years there was a continuous rise which, between 1919 and 1922, amounted altogether to 28 per cent. The Company's report cautiously states that it is too early as yet to say finally that the sudden check in the rising mortality from diabetes is to be credited to insulin. Before this can be done, figures must be obtained showing a decline for a series of years, and a greater rate of decrease must be shown. The rise between 1919 and 1922 may possibly have been due, in part at any rate, to an accumulation of cases which, but for the Great War, would have been spread over the years 1914–1919. But if the death-rate drops again in 1924, it will be safe to assume that there is some well-defined cause for the reversal of the upward tendency observed in 1919 and 1922. All that can be said at present is that no other outstanding factor likely to check the mortality from diabetes seems to have been operative during 1923.

### THE UNWORTHINESS OF INSECTS.

THE annual report of the Natural History Museum adds something to our knowledge of the unworthiness of insects. During the year there have been "very numerous" complaints of plagues of earwigs, which are commonly supposed to haunt chiefly the bottoms of flower-pots, but are now infesting districts which are being built upon. Then there is that cheerful and somewhat less unworthy creature, the cricket on the hearth, who is also becoming too numerous, and is colonising town refuse dumps. Unlike the sage advice about children, the cricket is heard rather than seen. Just as most young doctors have never seen a case of small-pox (thanks to exemptions they are likely to acquire experience in that direction), most respectable householders have seen a cricket even less often than they have seen a flea. Oddly enough, the report appears to be silent on the hideous subject of the domestic beetle, cockroach, or "black bat." It would be re-assuring to know that this terror which walks by night was being exterminated by the cricket, or even by the earwig. Benevolent naturalists have been known to suggest that insects have their uses, but an eager and incredulous world still awaits a clear and definite statement of what those uses are. Meanwhile the existence of the cockroach seems to be the clearest possible proof of the truth of evolution. That the Creator deliberately made the first cockroach is unthinkable. The race must assuredly have been evolved by the powers of evil working in their fiendish way for the affliction of mankind. It may quite conceivably spread disease as the house-fly does. We are ready to believe well of the pretty and attractive lady-bird, though even that may yet be proved to be a guilty creature; but the cockroach! The only persons whom it can ever have advantaged are those benefactors of the human race, Messrs. Keating, Blattis and Co.

### MALARIA IN AMERICA.

THE Treasury Department of the American Public Health Service has recently published (Public Health Bulletin, No. 137), the Transactions of the Fourth Annual Conference of Malaria Field Workers. The information contained in these 183 pages is remarkably varied. There is a highly scientific paper on the hydrogen-ion concentration of natural waters and the bearing this concentration may have on the distribution of mosquitoes, and there is a delightfully simple and entertaining paper, worded as a sort of catechism, in which the most elementary facts about malaria are concisely presented for the benefit of the school child and for those numerous adults whose intellects are elementary. One of the most interesting and promising papers in this collection is by Dr. C. P. Coogle, who has investigated the possibility of rendering houses distasteful to mosquitoes by spraying the buildings with creosote oil. The houses selected for this experiment were primitive one-room houses in the rural districts of the South where mosquitoes were prevalent. Before 25 houses were sprayed, three successive counts were made of the mosquitoes within them. All the household effects were then removed, and the walls and ceilings were sprayed. Approximately two gallons of creosote were used on each room, the price being only 12 to 15 cents per gallon. Even as long as ten weeks after this spraying, some of the houses were still found to be shunned by the mosquitoes, and this procedure would therefore seem to be well worth adopting on a large scale in the Southern States, where the coloured manual workers have hitherto been in the habit of burning rags, leather and feathers to secure a few hours' sleep and freedom from molestation. This volume gives a good bird's-eye view of the complexity of the administrative and educational problems connected with malaria throughout the United States. One of the greatest difficulties encountered seems to be keeping up public interest and co-operation.

### THE DRIED MILK REGULATIONS.

THESE regulations, which recently came into force, appear to be designed in the main to ensure that the public should know clearly what they are buying. It seems hardly necessary to state that the regulations do not interfere with the discretion of medical practitioners as to the quantities of dried milk which they are accustomed to advise to be supplied, but we do so because we understand from inquiries that there was some doubt about this. It is required that dried, partly skimmed milk should be so labelled with the addition of the words, " Should not be used for babies except under medical advice." A correspondent, writing to the *Lancet*, suggests that dried milk is at a disadvantage in having a prescribed standard of 3.6 per cent. of fat when the legal standard for wet milk is only 3 per cent. It seems worth while to point out that what exists in the case of wet milk is merely a presumptive standard ; if proceedings are taken for adulteration, the onus of proof is thrown on the prosecution if the milk contains more than 3 per cent. of fat, but rests on the defendant if there is less than 3 per cent. Attention is drawn in the *Lancet* to a matter of much more serious import. Infant foods, not sold as milk, which lack the essential constituents of a suitable food for infants, and in fact do a great deal of harm to them, can apparently be sold without restriction. This matter was made the subject of investigation and report to the Local Government Board in 1914, and restrictive provisions are clearly overdue.

### HOME HELPS.

NOTHING can be more dreary than the condition of a working class family deprived, through illness, of the active management of its mother. When a mother is laid up, cooking, cleaning, mending, washing and all the details of household routine become almost impossible of accomplishment unless a handy little daughter is kept away from school or a friendly neighbour comes to the rescue. While the family is forlorn, the sick mother, oppressed by the thought of confusion downstairs, finds it doubly hard to recuperate, and probably ends by taking up work again long before she should. She is thus less able to do that work efficiently, and her value as a national asset and as the mainstay of her home may become permanently impaired. In Birmingham the Corporation has realised the necessity of a sick mother being able to take her time over recovery, unharassed by worries or inadequate help, and the provision of a home help forms part of the Maternity and Child Welfare system. Twenty women—none of them dependent on their earnings—work on a rota system, and families using the helps are assessed according to their income at a small sum per day. Helps are also working in some London boroughs and in Liverpool, and now the Public Health Committee of Manchester has asked the City Council for a grant of £400 for the employment of two women as an experiment. This scheme, if widely adopted, would bring very real assistance to the working-class mother who bears uncomplainingly a particularly heavy burden.

### TRACKING DOWN EPIDEMICS OF THE TYPHOID GROUP.

THE detection of germs, like that of human criminals, requires the Sherlock Holmes touch, which, when crowned with success, is a legitimate cause for jubilant complacency. A recent incident in Denmark is certainly a feather in the cap of the sanitary detective and, incidentally, it shows that if a pure milk supply is to be guaranteed, the human purveyors, as well as the bovine producers of milk, should be kept under systematic medical supervision. In the autumn of 1923 several cases of paratyphoid fever were notified in a district where it had not existed for some time. The patients had consumed milk supplied by the same farm, which was, accordingly, paid a visit of inspection. It proved to be a model of its kind. The shed in which the forty-five cows were housed was well kept, and the cows themselves were in good condition, well cared for and without any sign of disease. All the inmates of the farm were also found to be well, and it seemed at first as if the hunt for a focus of infection would be futile. It appeared, however, that all the employees did not live on the farm. So with a perseverance deserving high praise, the inspecting visitors extended the scope of their investigations, and in a house situated on the other side of the main public road they at last ran their quarry to earth. Several children were found to be suffering from paratyphoid fever, and two of them were still in bed and feverish. The mother of one of them was a milkmaid employed on the farm, and her milking stools were found to harbour paratyphoid bacilli. She admitted that she had recently felt very ill, but had continued to milk morning and evening without notifying the farmer of her illness. At the farm itself it was the practice to boil all the milk consumed on the premises, and this was doubtless why no case of paratyphoid fever had developed in this household. New milkers being provided, and the milking utensils sterilised, the embargo on the farm's milk was withdrawn, and no further cases of paratyphoid fever developed among its customers.

